# NG2-glia: rising stars in stress-related mental disorders?

**DOI:** 10.1038/s41380-022-01838-7

**Published:** 2022-10-24

**Authors:** G. Poggi, Malin Wennström, M. B. Müller, G. Treccani

**Affiliations:** 1 Preclinical Laboratory for Translational Research into Affective Disorders, Department of Psychiatry, Psychotherapy and Psychosomatics, Psychiatric Hospital, University of Zürich, Zürich, Switzerland; 2 Clinical Memory Research Unit, Department of Clinical Sciences Malmö, Lund University, Malmö, Sweden; 3 Department of Psychiatry and Psychotherapy, University Medical Center of the Johannes Gutenberg-University Mainz, Mainz, Germany; 4 Leibniz Institute for Resilience Research, Mainz, Germany; 5 Institute of Anatomy, University Medical Center of the Johannes Gutenberg-University Mainz, Mainz, Germany

**Keywords:** Depression, Neuroscience

To the Editor:

For decades, the interest towards glia cells and their contribution to central nervous system (dys)function has been rising. This conceptual expansion opens new avenues to disentangle pathophysiological mechanisms underlying psychiatric illnesses. Amongst the glia cells, oligodendrocyte progenitor cells (OPCs)—also identified as NG2-glia—play a fundamental role in brain function and plasticity, via interaction with glial and neuronal cells, and modulation of neurotransmission [1]. Although in their initial stages, preclinical and clinical studies are identifying the involvement of OPC alterations in stress responding and stress-related psychiatric illnesses [2–7].

In a study published in this journal, Tanti et al. 2021 [8] analysed post-mortem samples from patients with major depression that experienced child abuse (MD-CA), a severe early life stressor predisposing to the development of psychiatric disorders [9], and compared them with samples from major depression patients that did not experience child abuse (MD-nCA) and with matched controls (CON). They identified increased density of perineuronal nets (PNNs) and increased proportion of parvalbumin (PV) neurons surrounded by PNNs in Broadman area (BA) 11–12 of MD-CA, compared to the respective areas in MD-nCA and CON. Intriguingly, the level of PNN-related transcripts in OPCs correlated with PNN density and it was elevated in MD-CA. PNN density also correlated with OPCs-PV neurons proximity and the latter was increased in MD-CA. Importantly, OPC and PV-neuron densities were both comparable amongst groups and therefore unlikely to mediate the observed effects.

Whilst providing evidence that CA leads to long-term changes in PNNs, at least in those individuals that have MD, the authors did not clarify how OPCs are implicated in this phenomenon. First, using a single-nucleus transcriptomics approach, the same group previously reported a significant dysregulation in gene expression in BA9 of major depressive (MD) patients compared to CON, independently of early life experiences. Many cell types were affected in MD, but the most prominent dysregulation was observed in the OPC transcriptome. However, in the present study only MD-CA displayed changes in PNN-mRNA in OPCs. If one now tries to reconcile these findings, the question of whether changes in OPC transcriptional profiles are dependent on region and previous aversive experiences will inevitably arise. Or does this further disclose the intrinsic heterogeneity of the OPC population? [10] In other words, is there an OPC subpopulation, specialised in the maintenance of PV interneuron and PNNs, that is particularly susceptible to early life stress? Indeed, it was recently reported that early life adversity (ELA) leads to long-lasting transcriptome changes in NG2-glia [2]. Considering the apparent interconnections between PNN-related gene expression in OPCs, PNN density and OPC-PV proximity, it is also tempting to speculate that spatial RNA sequencing might identify transcriptome signatures that could segregate OPCs based on their proximity to PV interneurons and on their involvement in PNNs maintenance. Critically, the identification of such sub-populations could provide novel candidates for cell-specific pharmacological interventions.

**Fig. 1:**
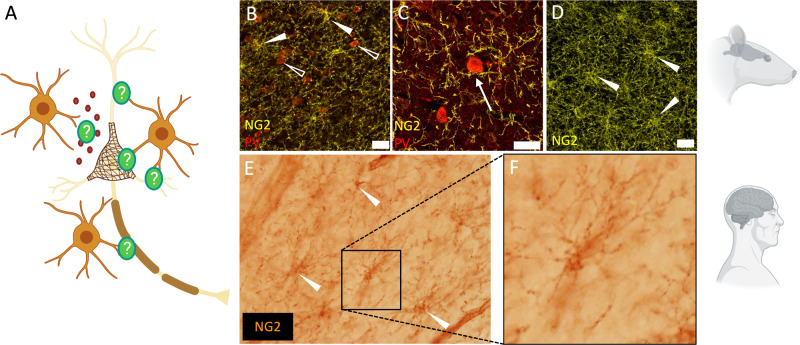
NG2-glia: morphology and proximity to PV-interneurons. **A** The image illustrates possible modalities of interaction between PV-interneurons and NG2-glia. **B** Confocal micrograph of NG2-glia (yellow, marked by full arrows) and PV^+^ interneurons (red, marked by empty arrows) in the mouse cortex, scale bar 20 um. Brightness and contrast have been adjusted for display purposes. **C** Confocal micrograph showing NG2-glia processes (yellow) in high proximity to the soma of PV-interneurons (red) in the mouse cortex, indicated by the arrow, scale bar 20 um. **D** Confocal micrograph showing the typical ramified morphology NG2-glia (yellow, marked by full arrows) in the mouse cortex, scale bar 20 um. Brightness and contrast have been adjusted for display purposes. **E**, **F** Representative staining that we performed with an anti-NG2 antibody (clone B5, ATCC, kind gift from Dr William Stallcup) in the hippocampus from human specimens obtained from the National Institute of Health (NIH) Bio Bank (USA). Brain specimens were obtained during autopsies conducted at the Allegheny County Medical Examiner’s Office (Pittsburgh, PA, USA) after consent for donation was obtained from the next-of-kin. All procedures were approved by the University of Pittsburgh’s Committee for the oversight of research and clinical training involving decedents and institutional review board for biomedical research. NIH NeuroBioBank approved the procedures of brain tissue collection, and the regional ethical review board in Lund, Sweden, approved the study (Dnr 2017/10). Figure was created with BioRender.com.

Second, the biological meaning of OPC-PV interneuron proximity should be explored beyond gene expression. Tanti et al. [8] labelled OPCs via in situ hybridization of *PDGFRa*, without counterstaining of the soma/processes, thus making it difficult to determine if, for instance, PV-OPC proximity entailed contact or might be purely related to paracrine functions exerted by NG2-glia on PNNs and PV interneurons (Fig. 1A–C). An immunostaining against an OPC marker, such as NG2, would be necessary to clarify this point (Fig. 1D, E). Immunostaining-based structural analysis could also reveal potential morphological changes in OPCs induced by exposure to early adversity. Indeed, reduced morphological complexity of OPCs was observed in the medial prefrontal cortex (mPFC) of adult mice exposed to chronic social defeat stress [7]. Here, whilst staining of NG2-glia in post-mortem tissues would be informative of OPC morphology and connections, technical difficulties in NG2-staining of human post-mortem tissues must be taken into consideration. For such staining, it is necessary that tissue fixation does not exceed 48 h and that the specimen is not embedded in paraffin [11]. Critically, this might also favour PV staining.

The role of NG2-glia in stress-related mental disorders is still controversial. Birey and colleagues [6] reported reduced OPC cell density in the frontal cortex of MD compared to CON, while Tanti et al. [8] did not. Also, another study led by Tanti [4] reported reduced density of OL lineage (Olig2^+^) cells in BA11, BA12 and BA32 of depressed suicide subjects with a history of CA (DS-CA) compared to CON [4]. This reduction did not pertain to OPCs and was associated with an increased density of mature oligodendrocytes (Nogo^+^ and APC^+^) [4], suggesting a shift of the OL lineage towards a more mature phenotype in DS-CA.

As well as post-mortem assessments in humans, preclinical studies provide heterogeneous results on the effect of stress on OPCs. In mPFC of mice exposed to ELA in the form of maternal separation (MS), NG2-glia increased and mature OLs (Olig2^+^/CC1^+^) decreased, compared to CON [12]. Proliferative NG2-glia also increased but did not differentiate into mature OLs, suggesting a deceleration of the OL lineage maturation dynamics [12]. In contrast, Teissier et al. [13] found that after MS, stressed pups presented with a reduced proliferation/cycling of OPCs and increased mature OLs. This resulted in the depletion of the OPC pool in adulthood [13]. In a different model of ELA, the limited bedding and nesting material paradigm, stress elicited corticosterone-induced changes in the transcriptome and increased voltage gated sodium channel-mediated currents distinctly in hippocampal NG2-glia [2]. In the adult mPFC, social defeat stress led to increased [14] and decreased [6] OPCs density in susceptible mice, while no changes in OPCs density were observed upon chronic social stress [3]. Reduction of NG2-glia proliferation was also observed in the mPFC of adult mice exposed to chronic stress [3, 7]. Interestingly, social defeat led to increased calcium signalling in hippocampal NG2-glia and the photoactivation of these cells elicited an anxiety-like phenotype in the mice in the absence of stress [15]. Taken together, findings to date strongly suggest changes in NG2-glia/OPCs in response to stress; these might be causally involved in stress-related psychiatric illnesses. Nevertheless, the causal mechanisms linking these phenomena still need to be elucidated. This will require in-depth characterization of the OPC population (or populations). Importantly, such characterization needs to take into account multiple variables, which might contribute to the inconsistency amongst published results, including type of stressor, time and duration of the stress exposure, brain region, and intrinsic OPCs heterogeneity. Dissecting the complex and, to some extent, still unknown biology of NG2-glia will provide understanding of their causal role in stress response and stress-related psychopathologies.
